# Supplementary material for: Complete Mitochondrial Genome Sequence and Identification of a Candidate Gene Responsible for Cytoplasmic Male Sterility in Celery (Apium graveolens L.)
Source: Int J Mol Sci. 2021 Aug 10;22(16):8584. doi: 10.3390/ijms22168584 (PMC8395238; doi:10.3390/ijms22168584)
Supplement: Supplementary file 1 [file ijms-22-08584-s001.zip › ijms-1271099-supplementary.pdf]

Supplementary Materials

Supplementary Figures

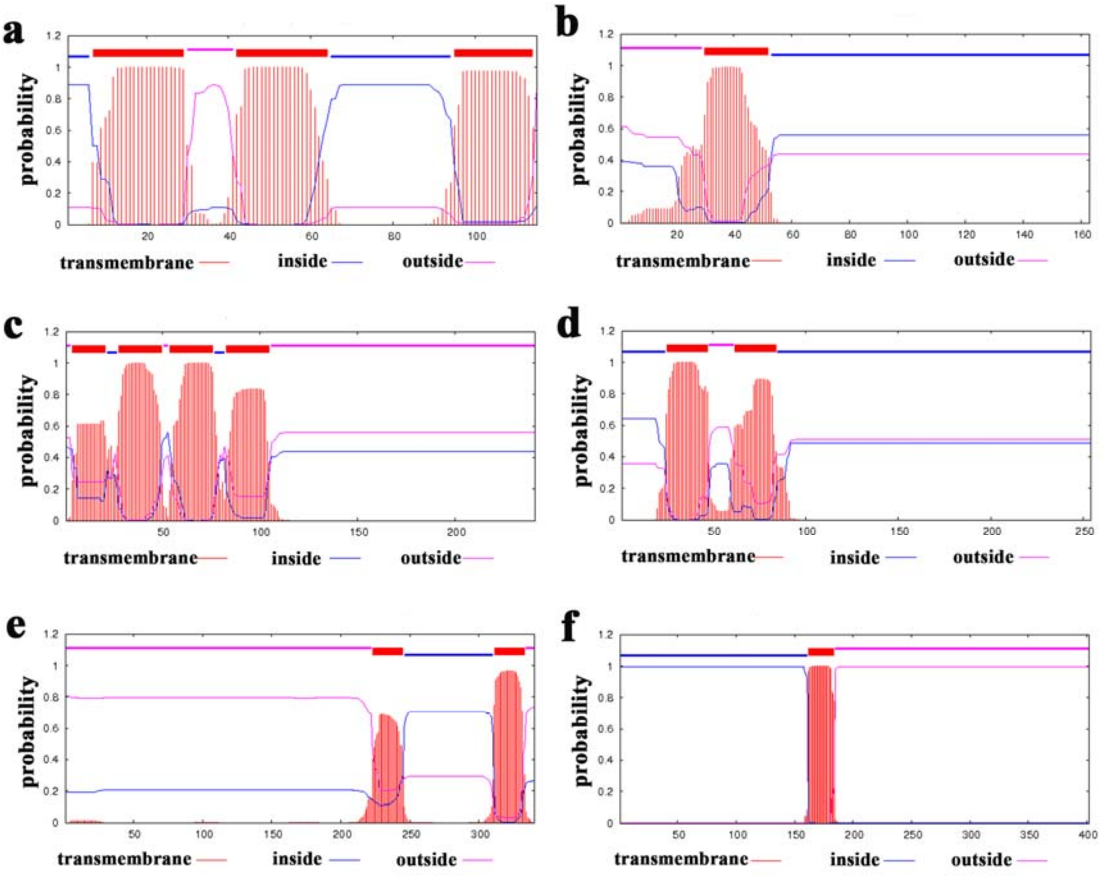

Supplementary Figure. S1 Transmembrane domains of the gene products of *orf115b* (a), *orf163a* (b), *orf241a* (c), *orf254a* (d), *orf340a-1/2* (e), *orf402a-1/2* (f).

Supplementary Tables

Supplementary Table S1 The results of genetic prediction of W99A and W99B

Gene ID in W99A	mRNA start (bp)	mRNA end (bp)	Gene ID in W99B	mRNA start (bp)	mRNA end (bp)
atp1	17037	18578	*atp1-1*	201090	202631
atp6	166366	167538	*atp1-2*	262114	263655
atp8	286217	286714	*atp1-3*	66905	68446
atp9-1	332585	332851	*atp6*	141486	142658
atp9-2	298149	298379	*atp8*	112607	113104
ccmB	164381	165001	*atp9*	355382	355648
ccmC-1	109117	109863	*ccmB-1*	40441	41061
ccmC-2	10359	11105	*ccmB-2*	174626	175246
ccmFc	176693	178820	*ccmB-3*	235650	236270
ccmFN	265589	267328	*ccmC*	147807	148553
cob	278732	279904	*ccmFc*	130205	132332
cox1	66205	67701	*ccmFN-1*	219332	221071

cox2	114012	117145	*ccmFN-2*	158308	160047
cox3	239684	240481	*cob-1*	232475	233647
matR	316421	318388	*cob-2*	171451	172623
mttB	263954	264676	*cox1-1*	91095	92591
nad1	119007	362586	*cox1-2*	286304	287800
nad2	159501	273084	*cox2*	17592	20724
nad3-1	366632	366988	*cox3*	37677	38474
nad3-2	40487	40843	*matR*	338016	339983
nad4-1	19743	28317	*mttB-1*	156673	157395
nad4-2	219242	227816	*mttB-2*	217697	218419
nad4L	96613	96885	*nad1*	22586	385382
nad5	101219	315199	*nad2-1*	164232	180126
nad6	181106	181723	*nad2-2*	225256	241150
nad7-1	347889	354112	*nad3*	389428	389784
nad7-2	53363	59585	*nad4-1*	69611	78185
nad9	125712	126284	*nad4-2*	264820	273394
orf101a-1	209224	209529	*nad4L-1*	283150	283422
orf101a-2	96623	96928	*nad4L-2*	87941	88213
orf101a-3	321389	321694	*nad5*	2463	354806
orf101b-1	81053	81358	*nad6*	127302	127919
orf101b-2	193654	193959	*nad7*	370687	376908
orf102a	127805	128113	*nad9*	29291	29863
orf103a-1	220055	220366	*orf100a*	117161	117463
orf103a-2	27193	27504	*orf101a-1*	87898	88203
orf103b	271103	271414	*orf101a-2*	283107	283412
orf103c	314693	315004	*orf101a-3*	342984	343289
orf103d-1	37544	37855	*orf101b*	328497	328802
orf103d-2	369620	369931	*orf102a*	31384	31692
orf103e	66576	66887	*orf103a-1*	77061	77372
orf104a-1	43445	43759	*orf103a-2*	272270	272581
orf104a-2	363716	364030	*orf103b-1*	163822	164133
orf108a	318859	319185	*orf103b-2*	224846	225157
orf108b	272365	272691	*orf103c*	336288	336599
orf109a-1	25277	25606	*orf103d*	392416	392727
orf109a-2	221953	222282	*orf103e-1*	91466	91777
orf109b-1	8271	8600	*orf103e-2*	286675	286986
orf109b-2	107029	107358	*orf104a*	386512	386826
orf112a-1	217796	218134	*orf104b*	148804	149118
orf112a-2	29425	29763	*orf106a-1*	38756	39076
orf113a	253673	254014	*orf106a-2*	172941	173261
orf113b	168713	169054	*orf106a-3*	233965	234285
orf115a-1	332853	333200	*orf107a*	202888	203211
orf115a-2	298381	298728	*orf108a*	340454	340780
orf115b	308836	309183	*orf108b-1*	165084	165410

orf115c	141746	142093	*orf108b-2*	226108	226434
orf115d	161229	161576	*orf108c*	146616	146942
orf116a	274432	274782	*orf109a-1*	75145	75474
orf116b	173702	174052	*orf109a-2*	270354	270683
orf118a-1	46339	46695	*orf109b*	8272	8601
orf118a-2	360780	361136	*orf111a-1*	62137	62472
orf121a-1	71364	71729	*orf111a-2*	196322	196657
orf121a-2	183965	184330	*orf111a-3*	257346	257681
orf121b-1	6551	6916	*orf112a-1*	79293	79631
orf121b-2	105309	105674	*orf112a-2*	274502	274840
orf122a-1	49709	50077	*orf113b*	139970	140311
orf122a-2	357398	357766	*orf115a*	355650	355997
orf122b-1	1910	2278	*orf115c*	307047	307394
orf122b-2	100668	101036	*orf115d-1*	43866	44213
orf122c	140143	140511	*orf115d-2*	178051	178398
orf122d	159811	160179	*orf115d-3*	239075	239422
orf123a	155650	156021	*orf116a-1*	167151	167501
orf124a	264302	264676	*orf116a-2*	228175	228525
orf124b	170428	170802	*orf116b*	134973	135323
orf126a	257394	257774	*orf117a*	393104	393457
orf128a-1	59199	59585	*orf118a*	383576	383932
orf128a-2	347889	348275	*orf121a*	124695	125060
orf128b	153057	153443	*orf121b*	6552	6917
orf129a-1	36816	37205	*orf122a*	380194	380562
orf129a-2	370270	370659	*orf122b*	1912	2280
orf129b	119007	119396	*orf122c*	308629	308997
orf132a	180147	180545	*orf122d-1*	45263	45631
orf133a-1	338875	339276	*orf122d-2*	179448	179816
orf133a-2	304403	304804	*orf122d-3*	240472	240840
orf133b-1	216901	217302	*orf122e*	148591	148959
orf133b-2	30257	30658	*orf123a-1*	183606	183977
orf134a-1	22520	22924	*orf123a-2*	49421	49792
orf134a-2	224635	225039	*orf123a-3*	244630	245001
orf134b	327278	327682	*orf124a-1*	157021	157395
orf134c	161312	161716	*orf124a-2*	218045	218419
orf137a	288160	288573	*orf124b*	138222	138596
orf1390a-1	81920	86092	*orf128a*	370687	371073
orf1390a-2	194521	198693	*orf128b-1*	51999	52385
orf139a-1	28504	28923	*orf128b-2*	186184	186570
orf139a-2	218636	219055	*orf128b-3*	247208	247594
orf142a-1	29801	30229	*orf129b*	22586	22975
orf142a-2	217330	217758	*orf132a*	128480	128878
orf142b	171792	172220	*orf132b-1*	192861	193259
orf143a-1	1962	2393	*orf132b-2*	58676	59074

orf143a-2	100720	101151	*orf132b-3*	253885	254283
orf146a	243946	244386	*orf133a*	361672	362073
orf147a-1	102678	103121	*orf133b-1*	80125	80526
orf147a-2	3920	4363	*orf133b-2*	275334	275735
orf148a-1	45758	46204	*orf134a-1*	72388	72792
orf148a-2	361271	361717	*orf134a-2*	267597	268001
orf149a	261828	262277	*orf134b*	350075	350479
orf154a-1	55828	56292	*orf134c-1*	43726	44130
orf154a-2	351183	351647	*orf134c-2*	177911	178315
orf159a-1	15054	15533	*orf134c-3*	238935	239339
orf159a-2	232026	232505	*orf1382a*	323787	327935
orf160a-1	37314	37796	*orf139a-1*	78372	78791
orf160a-2	369679	370161	*orf139a-2*	273581	274000
orf163a	306634	307125	*orf142a-1*	79669	80097
orf165a	287295	287792	*orf142a-2*	274878	275306
orf171a-1	26031	26546	*orf143a*	1964	2395
orf171a-2	221013	221528	*orf147a*	3922	4365
orf171b	112910	113425	*orf147b*	34146	34589
orf174a	118618	119142	*orf148a*	384067	384513
orf175a-1	27790	28317	*orf148b-1*	59675	60121
orf175a-2	219242	219769	*orf148b-2*	193860	194306
orf176a	114012	114542	*orf148b-3*	254884	255330
orf180a	114472	115014	*orf154a*	373979	374443
orf182a	341489	342037	*orf157a-1*	61342	61815
orf185a-1	3042	3599	*orf157a-2*	195527	196000
orf185a-2	101800	102357	*orf157a-3*	256551	257024
orf189a-1	183715	184284	*orf160a*	392475	392957
orf189a-2	71114	71683	*orf171a-1*	75899	76414
orf190a	68550	69122	*orf171a-2*	271108	271623
orf192a	340910	341488	*orf171b*	16490	17005
orf199a-1	212468	213067	*orf174a*	22197	22721
orf199a-2	34492	35091	*orf175a-1*	77658	78185
orf199a-3	1109	1708	*orf175a-2*	272867	273394
orf199a-4	99867	100466	*orf176a*	17592	18122
orf209a	243211	243840	*orf180a*	18052	18594
orf214a	161995	162639	*orf182a*	364286	364834
orf241a	295196	295921	*orf185a*	3044	3601
orf249a-1	10356	11105	*orf189a*	124741	125310
orf249a-2	109114	109863	*orf192a*	363707	364285
orf254a	309291	310055	*orf199a-1*	84360	84959
orf276a	320026	320856	*orf199a-2*	279569	280168
orf289a	177951	178820	*orf199a-3*	1109	1708
orf294a	176693	177577	*orf202a*	136805	137413
orf305a-1	280261	281178	*orf206a-1*	193377	193997

orf305a-2	238446	239363	*orf206a-2*	59192	59812
orf340a-1	189408	190430	*orf206a-3*	254401	255021
orf340a-2	76807	77829	*orf2105a*	113685	120002
orf343a-1	190423	191454	*orf214a-1*	42803	43447
orf343a-2	77822	78853	*orf214a-2*	176988	177632
orf399a	312904	314103	*orf214a-3*	238012	238656
orf402a-1	94882	96090	*orf249a*	147804	148553
orf402a-2	207483	208691	*orf276a*	341621	342451
orf417a-1	192326	193579	*orf289a*	130205	131074
orf417a-2	79725	80978	*orf294a*	131448	132332
orf529a-1	3448	5037	*orf305a-1*	10534	11451
orf529a-2	102206	103795	*orf305a-2*	106651	107568
orf768a	66205	68511	*orf305a-3*	301860	302777
orf810a-1	86022	88454	*orf319a-1*	105474	106433
orf810a-2	198623	201055	*orf319a-2*	300683	301642
rpl10	112937	113425	*orf334a-1*	195312	196316
rpl16-1	101800	102016	*orf334a-2*	61127	62131
rpl16-2	3042	3258	*orf334a-3*	256336	257340
rpl5-1	108519	109073	*orf347a-1*	88736	89779
rpl5-2	9761	10315	*orf347a-2*	283945	284988
rps1	294135	294740	*orf399a*	334499	335698
rps12-1	366206	366583	*orf417a*	328877	330130
rps12-2	40892	41269	*orf529a*	3450	5039
rps13-1	363457	363807	*orf958a*	320981	323857
rps13-2	43668	44018	*rpl10*	16517	17005
rps3-1	3457	5148	*rpl16*	3044	3260
rps3-2	102215	103906	*rpl5*	9762	10316
rps7	65377	65823	*rps1-1*	299684	300289
			rps1-2	104475	105080
			rps12	389002	389379
			rps13	386253	386603
			rps3	3459	5150
			rps7-1	90267	90713
			rps7-2	285476	285922

Supplementary Table S2 The specific orfs in W99A and their transmembrane domain number

No.	Gene ID	mRNA start (bp)	mRNA end (bp)	No.of transmembrane domain
1	*orf113a*	253673	254014	0
2	*orf115b*	308836	309183	3
3	*orf126a*	257394	257774	0
4	*orf129a-1*	36816	37205	0
	orf129a-2	370270	370659	0
5	*orf137a*	288160	288573	0
6	*orf142b*	171792	172220	1

7	*orf146a*	243946	244386	0
8	*orf149a*	261828	262277	0
9	*orf159a-1*	15054	15533	0
	orf159a-2	232026	232505	0
10	*orf163a*	306634	307125	1
11	*orf165a*	287295	287792	0
12	*orf190a*	68550	69122	3
13	*orf209a*	243211	243840	0
14	*orf241a*	295196	295921	4
15	*orf254a*	309291	310055	2
16	*orf340a-1*	189408	190430	2
	orf340a-2	76807	77829	2
17	*orf343a-1*	190423	191454	0
	orf343a-2	77822	78853	0
18	*orf402a-1*	94882	96090	1
	orf402a-2	207483	208691	1
19	*orf768a*	66205	68511	11
20	*orf810a-1*	86022	88454	0
	orf810a-2	198623	201055	0
21	*orf1390a-1*	81920	86092	0
	orf1390a-2	194521	198693	0

Supplementary Table S3 The specific ORFs in W99B and their transmembrane domain number

No.	Gene ID	mRNA start (bp)	mRNA end (bp)	No.of transmembrane domain
1	*orf100a*	117161	117463	0
2	*orf104b*	148804	149118	0
3	*orf106a-1*	38756	39076	1
	orf106a-2	172941	173261	1
	orf106a-3	233965	234285	1
4	*orf107a*	202888	203211	1
5	*orf108c*	146616	146942	0
6	*orf111a-1*	62137	62472	2
	orf111a-2	196322	196657	2
	orf111a-3	257346	257681	2
7	*orf117a*	393104	393457	0
8	*orf122e*	148591	148959	1
9	*orf132b-1*	192861	193259	0
	orf132b-2	58676	59074	0
	orf132b-3	253885	254283	0

10	*orf1382a*	323787	327935	0
11	*orf147b*	34146	34589	0
12	*orf148b-1*	59675	60121	0
	orf148b-2	193860	194306	0
	orf148b-3	254884	255330	0
13	*orf157a-1*	61342	61815	0
	orf157a-2	195527	196000	0
	orf157a-3	256551	257024	0
14	*orf202a*	136805	137413	2
15	*orf206a-1*	193377	193997	0
	orf206a-2	59192	59812	0
	orf206a-3	254401	255021	0
16	*orf2105a*	113685	120002	1
17	*orf319a-1*	105474	106433	3
	orf319a-2	300683	301642	3
18	*orf334a-1*	195312	196316	2
	orf334a-2	61127	62131	2
	orf334a-3	256336	257340	2
19	*orf347a-1*	88736	89779	1
	orf347a-2	283945	284988	1
20	*orf958a*	320981	323857	0

Repeat Length	W99A			W99B		
	Number	Bases	Coverage %	Number	Bases	Coverage %
30-100	73	5830	1.57	67	4657	1.18
101-300	18	2608	0.70	25	5240	1.33
301-500	0	0	0.00	0	0	0.00
501-1000	0	0	0.00	0	0	0.00
1001-2000	7	13233	3.56	9	15873	4.03
2001-5000	7	29788	8.02	8	41252	10.47
5001-10000	4	30326	8.17	2	12442	3.16
10001-20000	2	23572	6.35	0	0	0.00
20001-30000	4	107371	28.92	2	59782	15.17
30001-40000	2	63834	17.19	0	0	0.00
40000+	0	0	0.00	4	247048	62.69
Total	117	241433*	65.03	117	284284*	72.14

Note: Repeats were identified by blasting each genome to itself (ncbi-blast-2.2.30+; Similarity ≧ 97% and length ≧ 30 bp). Repeats with identical begin and end coordinates are counted only once. Percent coverage is the percentage of nucleotide positions in the genome occupied by repeats of given length category, calculated without respect to repeats in other length categories. * means the whole repeat sequence, calculated without the overlap repeats in other given length category.

Supplementary Table S5 The location information of W99A unique regions and the orfs in it

No.	start (bp)	end (bp)	size(bp)	orf name	orf start(bp)	orfend(bp)	No. of transmembrane domain
1	11381	16192	4812	*orf159a-1*	15054	15533	0
2	67689	68526	838	*orf768a*	66205	68511	11
3	75520	76054	535	*NA*			
4	76810	79379	2570	*orf340a-2*	76807	77829	2
				orf343a-2	77822	78853	0
5	89267	90032	766	*NA*			
6	90944	95658	4715	*orf402a-1*	94882	96090	1
7	110139	110404	266	*NA*			
8	143340	145344	2005	*NA*			
9	147068	147512	445	*NA*			
10	148948	149293	346	*NA*			
11	188121	188655	535	*NA*			
12	189411	191980	2570	*orf340a-1*	189408	190430	2
				orf343a-1	190423	191454	0
13	201868	202633	766	*NA*			
14	203545	208259	4715	*orf402a-2*	207483	208691	1

15	231367	235772	4406	*orf159a-2*	232026	232505	0
16	243251	246821	3571	*orf209a*	243211	243840	0
				orf146a	243946	244386	0
17	252734	256048	3315	*orf113a*	253673	254014	0
18	256294	262523	6230	*orf126a*	257394	257774	0
				orf149a	261828	262277	0
19	288124	288305	182	*orf137a*	288160	288573	0
20	294831	297212	2382	*orf241a*	295196	295921	4
21	306467	310583	4117	*orf163a*	306634	307125	1
				orf115b	308836	309183	3
				orf254a	309291	310055	2

Supplementary Table S6 The location information of W99B unique regions and the orfs in it

No.	start (bp)	end (bp)	size(bp)	orf name	orf start(bp)	orfend(bp)	No.of transmembrane domain
1	10374	10525	152	*NA*			
2	33087	34907	1821	*orf147b*	34146	34589	0
3	38793	39101	309	*orf106a-1*	38756	39076	1
4	56149	66046	9898	*orf132b-2*	58676	59074	0
				orf206a-2	59192	59812	0
				orf148b-1	59675	60121	0
				orf334a-2	61127	62131	2
				orf157a-1	61342	61815	0
				orf111a-1	62137	62472	2
5	89168	90118	951	*orf347a-1*	88736	89779	1
6	92579	92758	180	*NA*			
7	105171	106642	1472	*orf319a-1*	105474	106433	3
8	114506	120905	6400	*orf2105a*	113685	120002	1
				orf100a	117161	117463	0
9	142793	147760	4968	*orf108c*	146616	146942	0
10	148829	151554	2726	*orf122e*	148591	148959	1

				orf104b	148804	149118	0
11	151800	153819	2020	*NA*			
12	172978	173286	309	*orf106a-2*	172941	173261	1
13	190334	200231	9898	*orf132b-1*	192861	193259	0
				orf206a-1	193377	193997	0
				orf148b-2	193860	194306	0
				orf334a-1	195312	196316	2
				orf157a-2	195527	196000	0
				orf111a-2	196322	196657	2
14	202654	203472	819	*orf107a*	202888	203211	1
15	204384	212578	8195	*NA*			
16	212824	214843	2020	*NA*			
17	234002	234310	309	*orf106a-3*	233965	234285	1
18	251358	261255	9898	*orf132b-3*	253885	254283	0
				orf206a-3	254401	255021	0
				orf148b-3	254884	255330	0
				orf334a-3	256336	257340	2
				orf157a-3	256551	257024	0
				orf111a-3	257346	257681	2
19	284377	285327	951	*orf347a-2*	283945	284988	1
20	287788	287967	180	*NA*			
21	300380	301851	1472	*orf319a-2*	300683	301642	3
22	305358	305412	55	*NA*			
23	305778	305800	23	*NA*			
24	320546	320589	44	*NA*			
25	320984	321373	390	*orf958a*	320981	323857	0
26	330476	332178	1703	*NA*			

Supplementary Table S7 The results of SNP annotation with W99A as the reference sequence

Sample ID	CDS					Intergenic	Total
	Start-nonsyn	Stop-nonsyn	Premature-stop	Synonymous	Nonsynonymous		
W99B	0	0	1	4	9	55	69

Note: Start_nonsyn: non-synonymous mutations of start codon; Stop_nonsyn: non-synonymous mutation of termination codon; Premature_stop: nonsense mutation, the triplet codon mutates into a stop codon; Synonymous: synonymous mutation in the gene region; Nonsynonymous: non-synonymous mutations in the gene region; Intergenic：SNP in the intergenic region

Supplementary Table S8 The results of InDel annotation with W99A as the reference sequence

Sample ID	I-gene-middle	D-gene-middle	Intergenic	Total
W99B	1	2	6	9

Note: I-gene-middle：insertion in CDS; D-gene-middle: deletion in CDS; Intergenic：Intergenic: InDel in the intergenic region.

Table S9 Sequence variations of mitochondrial genes coding for known and hypothetical proteins between W99A and W99B

Gene ID	position	Mutation type	(W99B→W99A) Base mutation	(W99B→W99A) amino acid mutation
orf165a	287549	SNP	G→T	V→F
orf71f	241726	SNP	T→G	I→R
	241737	SNP	A→C	L→F
cob	279301	SNP	A→C	I→L
orf174a	119003	SNP	A→C	Y→S
orf182a	341866	SNP	A→C	M→L
orf87g	127357	SNP	A→G	K→R
orf90b	180675	SNP	A→C	L→F
rps1	294157	SNP	C→G	A→G
orf88k	65915	SNP	T→G	X→E
orf129a-2	370270	InDel	Insertion	frame-shifted
orf91b	57040	InDel	Deletion	frame-shifted
orf76g	139910	InDel	Deletion	frame-shifted

Supplementary Table S10 Sequence variations of *orf768a* and *cox1*

Mutant base position(bp)	*cox1→orf768a* Base mutation	*cox1→orf768a* Codon mutation	*cox1→orf768a* amino acid mutation	mutation results
1485	C→A	GGC→GGA	G→G	Synonymous
1486	T→A	TGT→ AGA	C→R	Nonsynonymous
1488	T→A			
1489	G→A	GTA→AAG	V→K	Nonsynonymous
1490	T→A			
1491	A→G			
1492	A→G	AGT →GGC	S→G	Nonsynonymous
1494	T→C			
1495	T→G	TAA→GAA	stop codon→E	

Supplementary Table S11 Detection of six CMS-associated candidate orf gene markers

molecular marker	Accuracy (%)	molecular marker	Accuracy (%)
orf115b	23	*orf340a-1/2*	22
orf163a	25	*orf402a-1/2*	82

orf254a	24	*orf768a*	100

Supplementary Table S12 Validity test of the orf768a marker in 41 breeding lines

Sample number	Sample name	Phenotype	orf768a genotype	Sample numbe	Sample ID	Phenotype	orf768a genotype
1	18Q100	normal	normal	22	18Q 181	normal	normal
2	18Q135	CMS	CMS	23	18Q 182	normal	normal
3	18Q142	normal	normal	24	18Q 183	CMS	CMS
4	18Q146	normal	normal	25	18Q 189	normal	normal
5	18Q147	CMS	CMS	26	18Q 198	normal	normal
6	18Q148	normal	normal	27	18Q 203	normal	normal
7	18Q149	CMS	CMS	28	18Q 208	normal	normal
8	18Q151	normal	normal	29	18Q 210	normal	normal
9	18Q153	normal	normal	30	18Q 212	normal	normal
10	18Q155	normal	normal	31	18Q 214	normal	normal
11	18Q157	normal	normal	32	18Q 215	normal	normal
12	18Q158	CMS	CMS	33	18Q 216	normal	normal
13	18Q159	normal	normal	34	18Q 222	normal	normal
14	18Q162	normal	normal	35	18Q 224	normal	normal
15	18Q172	normal	normal	36	18Q 228	normal	normal
16	18Q173	CMS	CMS	37	18Q 229	CMS	CMS
17	18Q174	normal	normal	38	18Q 230	normal	normal
18	18Q176	normal	normal	39	18Q 231	CMS	CMS
19	18Q177	CMS	CMS	40	18Q 232	normal	normal
20	18Q179	normal	normal	41	18Q 265	normal	normal
21	18Q180	CMS	CMS				

Supplementary Table S13 List of primers in this study

**Primer names**	**Forward (5'– 3')**	**Reverse (5'– 3')**	**Purpose**
Corf115b	ATGCCTATAATAAGAAAAAGCACG G	TCAACTCATATACAAGAAAATCGATAC AG	RT-PCR/marker
Corf163a	ATGATACACAGCCCACTTGAGCA	TCATACACCACATTGCCGTCC	RT-PCR/marker
Corf241a	ATGCACAGAGGAAGAGAACGAAC	CTACCCAGAAGCAGAAGGTCCAG	RT-PCR
Corf254a	ATGTGGAGCCTATCCCCTCAGC	TCATCTGTTGTTTTGGTTTTCTTCCC	RT-PCR/marker

Corf340a-1/2	ATGTTCTGTTCATATATGTCTGTTGA	CTATACATTGTTCATTACATTACTCATATC	RT-PCR/marker
Corf402a-1/2	ATGCACAGAGGAAGAGAACGAAC	TTACCCCGTCCCGTCTCC	RT-PCR/marker
AgACTIN**(GeneID：101260631)**	CTTCCTGCCATATATGATTGG	GCCAGCACCTCGATCTTCATG	RT-PCR
